# Outcomes of Laparoscopic Cesarean Scar Defect Repair: Retrospective and Observational Study

**DOI:** 10.3390/jcm12113720

**Published:** 2023-05-28

**Authors:** Camran Nezhat, Benjamin Zaghi, Kelly Baek, Azadeh Nezhat, Farr Nezhat, Steven Lindheim, Ceana Nezhat

**Affiliations:** 1 Camran Nezhat Institute, Palo Alto, CA 94061, USA; 2 Stanford University Medical Center, Stanford, CA 94305, USA; 3 University of California, San Francisco, CA 94143, USA; 4 California Fertility Partners, Los Angeles, CA 90025, USA; drkellybaek@californiafertilitypartners.com; 5 Nezhat Surgery for Gynecology/Oncology, New York, NY 10128, USA; 6 Department of Obstetrics and Gynecology, NYU Hospital, NYU Long Island School of Medicine, Mineola, NY 10016, USA; 7 Department of Obstetrics and Gynecology, Weill Cornell Medical College of Cornell University, New York, NY 10065, USA; 8 Department of Obstetrics and Gynecology, University of Central Florida, Orlando, FL 32827, USA; 9 Boonshoft School of Medicine, Wright State University, Dayton, OH 45324, USA; 10 Nezhat Medical Center, Atlanta Center for Minimally Invasive Surgery and Reproductive Medicine, Atlanta, GA 30342, USA

**Keywords:** niche, isthmocele, cesarean scar defect, uteroperitoneal fistula, uterine diverticulum, pregnancy

## Abstract

Cesarean scar defect, also known as niche, isthmocele, uteroperitoneal fistula and uterine diverticulum, is a known complication after cesarean delivery. Due to the rising cesarean delivery rates, niche has become more common and can present as irregular bleeding, pelvic pain, infertility, cesarean scar pregnancy and uterine rupture. Treatments for symptomatic cesarean scar defect vary and include hormonal therapy, hysteroscopic resection, vaginal or laparoscopic repair, and hysterectomy. We report on the safety and efficacy of our method of repairing cesarean scar defects in 27 patients without adverse outcomes: two-layer repair where the suture does not enter the uterine cavity. Our method of laparoscopic niche repair improves symptoms in nearly 77% of patients, restores fertility in 73% of patients, and decreases the time to conception.

## 1. Introduction

Cesarean section rate has significantly increased to 30% over the last decade, resulting in abnormal placentation such as accreta, scar dehiscence or uterine rupture, as well as cesarean scar pregnancy [1,2]. In 1961, Dr. Poidevin, from Australia, was the first to associate a uterine scar defect detected via hysterosalpingography with “explosive or complete uterine rupture” [3]. The prevalence of cesarean scar defect ranges from 19% to 61% after one cesarean and could be as high as 100% in women after three cesareans. These numbers might be underestimated due to women being asymptomatic as well as the lack of awareness among practitioners [4,5,6,7]. The cesarean scar defect is also known as niche, uteroperitoneal fistula, uterine diverticulum and isthmocele. In 1995, Dr. Hugh Morris studied 51 hysterectomy specimens and demonstrated that the niche was associated with menorrhagia, dyspareunia and dysmenorrhea. Unfortunately, he was not given much attention. Improper cesarean incision healing leads to a defect on the anterior uterine wall.

Multiple risk factors for niche have been reported. A recent study demonstrated that higher maternal body mass index, gestational diabetes and previous cesarean deliveries were all associated with an increased risk for incomplete healing of the uterine incision. This study evaluated 371 women with a sonohysterogram six months after cesarean delivery, and a niche was found in 45% of these patients, regardless of whether the cesarean delivery was emergent or elective. For every additional unit of body mass index (BMI) increase, the risk of niche increased by 6% [8,9]. Other risk factors included labor prior to cesarean section leading to a low hysterotomy and uterine closure as a single-layer or locking sutures, as well as peritoneal non-closure [10]. As such, the niche can lead to postmenstrual spotting, pelvic pain, vaginal discharge, dysmenorrhea, dyspareunia, infertility and obstetrical complications, including abnormal placentation and uterine rupture. It can also pose a greater risk for complications during an intrauterine device insertion, endometrial ablation and hysterectomy due to the proximity of the scar to the bladder [11]. The most reported test for identifying the niche is a transvaginal ultrasonogram (TVS), which can appear as a wedge defect, protrusion of the scar, hematoma, triangular anechoic area, or debris-filled cystic mass between the bladder and lower uterine segment [12,13,14,15,16]. 

Currently, there are three described methods for surgical repair of the defect: hysteroscopic resection, laparoscopic resection and repair (with or without robotic assistance) and vaginal repair. Our technique includes a two-layer closure without having the suture enter the uterine cavity. While we advised our patients to wait up to 6 months before trying to conceive, many patients conceived spontaneously and sooner than we had advised; some as early as 6 weeks. Without a change in adverse outcomes, such as uterine rupture or cesarean scar ectopic, we began counseling our patients to attempt conception as soon as 6 weeks post-procedure. The purpose of our retrospective study is to demonstrate the safety and efficacy of our technique: a two-layer niche repair without having the suture enter the uterine cavity and the clearance to attempt conception 6 weeks post-procedure. 

## 2. Materials and Methods

This study was approved by our local Institutional Review Board on 28 February 2023 (IRB Number: 20230726). All the patients underwent surgical intervention for niche between March 2015 and July 2019. Patients with infertility were referred to us for niche repair after they had been evaluated and treated by their infertility specialists. Additionally, the patients who underwent hysterectomy had already completed family planning, and they were referred to our center for hysterectomy due to pain and/or irregular vaginal bleeding.

**Figure 1:**
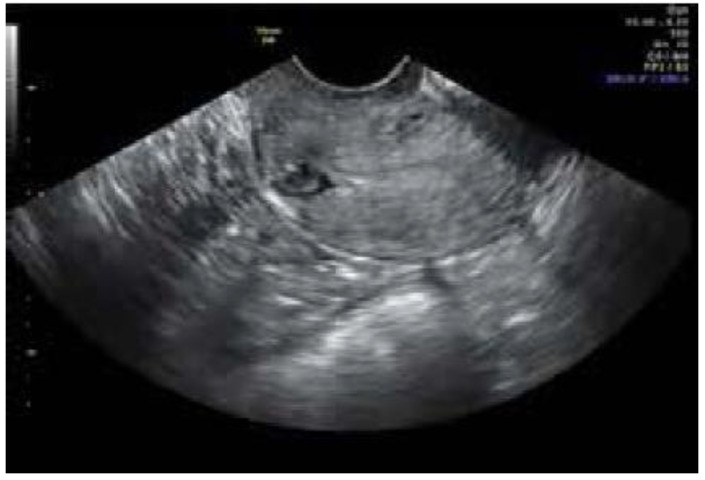
Transvaginal ultrasound can be used for in-office diagnosis. Ultrasound courtesy of Antony Dobson, MD, PhD.

**Figure 2:**
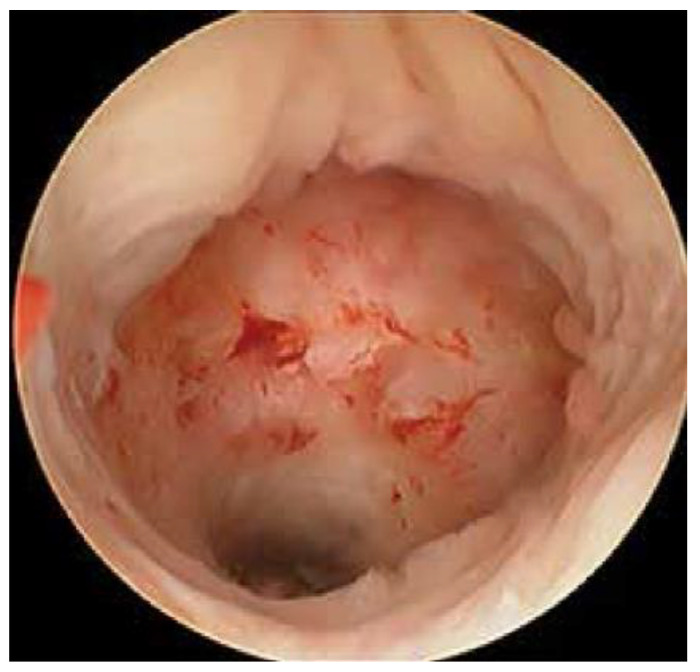
A hysteroscopic view of a niche. Notice the outpouching into the anterior lower uterine segment.

We included patients with symptomatic niche with a diagnosis made by transvaginal ultrasound, saline sonogram, or pelvic MRI, and confirmed via hysteroscopy, as demonstrated in Figure 1 and Figure 2. Those excluded were patients who had any congenital uterine anomalies.

Our surgical protocol was as follows: With the patient under general anesthesia and in a dorsal lithotomy position, the bladder is emptied. Video hysteroscopy is then performed to confirm and visualize the defect. The abdomen is entered via a video laparoscope. The light from the video hysteroscope is easily visualized laparoscopically by the thinned myometrium in the area of the defect. At this point, a bladder flap is developed by reverse vesicouterine fold dissection technique to mobilize the bladder inferiorly, as described previously [17]. This technique is ideal for patients with dense adhesions between the bladder and the lower uterine segment. Using an inferior to superior sweeping motion with a blunt probe, the bladder is dissected off the uterus from an unscarred plane.

Transcervically, a video hysteroscope is passed to map out the niche via transilluminating the thinned myometrium, which can be visualized laparoscopically. Care must be taken when inserting the device into the niche, as there is often overlying bladder. One can prevent incidental cystotomy by gently advancing the video hysteroscope into the defect until the niche can be adequately detected. After the niche has been mapped, the video hysteroscope is removed, and a cervical dilator or uterine manipulator is gently introduced into the niche. With the guiding instrument denoting the area of the defect, the fibrotic edges of the niche are excised preferentially with sharp dissection, which leaves healthy myometrial tissue margins. These margins are reapproximated with a delayed-absorbable (2-0 polyglactin 910 [Vicryl]) or barbed suture (2-0 V-Loc) in a running, non-locked fashion using a two-layer closure, with careful attention to reapproximate the myometrium-myometrial edges without entering the uterine cavity, followed by serosal-serosal edges. Following the video laparoscopic repair, a video hysteroscopic evaluation of the uterine cavity is again performed to ensure complete resolution of the defect, as well as to confirm that no sutures are within the cavity. Based on our experience, exposed suture within the cavity can lead to adhesion formation. With the video hysteroscope in place, the laparoscopic assessment confirms that the repair is satisfactory when no hysteroscopic fluid escapes through the repaired hysterotomy. Chromopertubation is also performed concurrently to assess for tubal patency and to ensure the repair is complete.

Statistical analysis: Mean ± standard deviation was used to describe normally distributed continuous data. Median was used to describe non-normally distributed continuous data. 

## 3. Results

A total of 27 patients underwent surgical treatment for symptomatic niche during the study period and their characteristics are summarized in Table 1. The most common presenting symptom was pelvic pain (81%), dysmenorrhea (70%), irregular vaginal bleeding (67%), followed by infertility (14%), and urinary symptoms (44%). Twenty patients had undergone one previous cesarean delivery, six had undergone two cesarean deliveries and one had undergone four cesarean deliveries. None reported a history of c-section scar ectopic. The duration of infertility was more than 1 year in 40% of our patients. Prior to coming to our center, 52% attempted in vitro fertilization (IVF) while 29.6% attempted intrauterine insemination (IUI). In total, 44% of the patients had previously been diagnosed with endometriosis.

Most patients underwent laparoscopic repair (85.2%) compared with hysterectomy (11.1%) as well as hysteroscopic-only repair (3.7%). All the patients had confirmed niche on hysteroscopy.

The mean age at the time of surgical intervention was 36 (27–45 years). Of the patients, 11 (40%) had undergone previous surgery for the treatment of endometriosis. The clinical follow-up period ranged between 1 month to 3 years. There were two post-operative complications: one surgical site infection and one umbilical hernia requiring repair. 

Obstetric outcomes are summarized in Table 2. A total of 15 (55.6%) desired future fertility. The pregnancy rate after repair was 73% (11) with 60% (9) resulting in live birth. Nine conceived naturally and two conceived via IVF. There were two miscarriages. No serious complications were reported amongst the group that delivered, including cesarean scar pregnancy or uterine rupture, following our method of repair. 

The most common pathologic diagnosis of the resected scar was fibrotic tissue. Additionally, 67% (*n* = 18) had the presence of endometriosis in other pelvic areas. Symptoms were resolved in 33% of our patients, improved in 44%, and persisted in 18%. The other 5% failed to provide feedback. The wide range of follow-up could be explained by our center being a referral center and all the patients not necessarily following up, may have followed up with their referring physicians instead. Overall, 78% (*n* = 21) of patients noted a significant improvement or resolution of their primary complaint(s).

## 4. Discussion

As noted earlier, cesarean delivery rate remains high despite the recommendations from current Ob/Gyn guidelines set by ACOG [18]. The future repercussions of cesarean section include the spectrum of abnormal placentation, cesarean scar pregnancies, and uterine rupture [1,2]. In a prospective cohort study of 263 women, Van der Voet et al. found that the prevalence of niche 6–12 weeks after a cesarean was dependent on the mode of detection: detection via transvaginal sonogram was 49% and 64.5% via gel instillation sonohysterography [11]. Niche was thought to be a rare manifestation of infertility; however, we continue to see an increase in diagnosis and association, most likely because of a heightened awareness of the condition [19]. Women who had cesarean section were found to have a 9% lower subsequent pregnancy rate than those who had delivered vaginally [20].

Symptomatology of the niche includes abnormal bleeding, pelvic pain, vaginal discharge, dysmenorrhea and dyspareunia [21,22,23]. The presence of niche could also pose a greater risk for complications during an intrauterine device insertion, endometrial ablation and hysterectomy due to the close scar proximity to the bladder [10]. The niche can easily be visualized by video hysteroscopy as a thin, concave indentation of the myometrium in the lower uterine segment. The defect also could be found at the endocervical canal and mid-uterine body, depending on the site of the hysterotomy incision.

As the exact pathophysiology is not yet determined, there are several hypotheses based on infertility, patient-related factors and symptomatology. An inadequate surgical technique for a uterine incision closure could lead to improper scar healing, seen as a defect on the anterior uterine wall. Ischemic necrosis of the myometrial edges due to tight and locking sutures could lead to niche formation. Additionally, an inflammatory reaction could result in the formation of adhesions between the cesarean incision and nearby structures, such as the anterior abdominal wall, bladder and intestines. Adhesions have also been shown to form when the peritoneum is not closed [24]. Proponents of a non-peritoneal closure technique argue for shorter surgical time, shorter hospital stay and lower pain scores. However, these studies evaluated pain as their primary focus, which is very subjective [25]. The primary outcome should concentrate on post-operative complications such as adhesion formation and wound infection, all of which could lead to chronic pelvic pain, intestinal obstruction and infertility [26]. A postpartum uterus takes approximately six weeks to return to its position in the pelvis; however, the peritoneum heals within five days after a cesarean section [24]. If the peritoneum is not closed, the uterus could attach itself to the anterior abdominal wall, bladder, omentum and/or intestines, which is commonly seen in patients with a niche [24]. A study by Seyam et al. showed that patients whose peritoneum was reapproximated had fewer adhesions and higher pregnancy rates (40.2%) compared to the non-closed group (8.8%) [27].

Other factors that could decrease proper healing of the hysterotomy and therefore lead to a niche formation include prolonged labor, advanced cervical dilatation, oxytocin-induced labor, or the presence of a retroverted uterus [24,25,26,28]. Fibrotic tissue in the niche forms a pouch or reservoir for menstrual blood. This would enable menstrual blood to be trapped in this niche pouch and affect implantation by creating a toxic environment for sperm and embryo [4,27,29]. It is well-established that hydrosalpinx can lead to infertility as the fluid in the tube can disrupt implantation. The toxic environment of the niche has been compared to patients with hydrosalpinx. A niche may cause delayed menstruation through the cervix, resulting in abnormal bleeding, pelvic pain, vaginal discharge and dysmenorrhea. In patients with infertility, the site of the niche can have abnormal blood vessels and inflammation with decreased contractility of the lower uterine segment.

The preferred treatment approach remains controversial. Surgical repair should be indicated for symptomatic women as well as asymptomatic women with infertility and an obvious defect. The results of different repair techniques are mixed. This great variability can be attributed to studies without clear definition of population, without correction for confounders and without inclusion or exclusion criteria.

In symptomatic women, the outcomes of hysteroscopic repair range from 59% to 100%, while laparoscopic repair range from 63% to 86% [30]. Fertility rates range from 77% to 100% after hysteroscopic treatment, while after laparoscopic repair 23 to 75% [20,22,31,32,33]. Finally, fertility rates after vaginal repair have been reported to be 22% [30]. We found that 11 (73%) out of 15 patients who desired fertility were able to achieve pregnancy. This high rate could possibly be explained by pre-existing and diagnosis of endometriosis. It is known that endometriosis is found in up to 40% of infertile women, while 87% in women with chronic pelvic pain [34]. The coexistence of niche and endometriosis was only reported in two studies, 21.1–27.2% [4,20]. We found such coexistence in 67% of the patients while identifying 22% (*n* = 6) new cases. Ignoring the diagnosis and treatment of endometriosis during surgical intervention for niche repair can potentially result in suboptimal outcomes. Direct visualization allows accurate diagnosis of endometriosis as well as excision of the defect and reapproximation of the myometrial defect using a two-layer closure.

Our technique of niche repair utilized a two-layer closure without suture entering the uterine cavity. Previously, we advised our patients to wait six months prior to conceiving, however, many patients achieved pregnancy much earlier without any adverse outcomes. Thus, we now instruct them to start conceiving as early as 6 weeks. As reported above, we did not observe a difference in adverse outcomes or complications, including uterine rupture or cesarean scar pregnancy.

As first reported by Nezhat et al. in 2003, our experience over the past two decades, for women who desire future fertility, a laparoscopic approach is recommended [35]. Direct visualization allows accurate excision of the defect and re-approximation of the myometrium using a two-layer closure, without entering the uterine cavity. Furthermore, it allows diagnosis and treatment of endometriosis, which could also lead to pain, infertility and miscarriage. Studies report a residual myometrial thickness of less than 2 mm as an indicator for the uterine rupture during a trial of labor after cesarean delivery [36]. However, Tanimura et al. further stratified uterine retroflexion as well as residual myometrial thickness less than 2.5 mm and recommended video laparoscopic repair [20].

For those who have completed childbearing, we recommend video hysteroscopic approach if the myometrial thickness is 3 mm or greater to avoid bladder injury. Raimondo et al. reported 120 cases of hysteroscopic repair due to postmenstrual bleeding and pain. The procedure duration averaged a brief eight minutes with resolution of symptoms in 80% of patients. However, Donnez et al. reported the risk of bladder injury and uterine perforation greatly increases if the myometrium thickness at the defect site is <3 mm [4]. We similarly advocate for video laparoscopic excision and repair in the case of thin myometrium. In contrast, vaginal repair is associated with longer surgical time and greater blood loss compared to hysteroscopic repair; however, improvement in abnormal bleeding was significantly higher (93.5% vs. 64.5%) [30].

Lastly, hysterectomy is another option for patients who have completed childbearing. Ultimately, the surgical approach should be tailored to the future objective of the patient, whether it be pain or bleeding resolution, future fertility, or definitive treatment. Being familiar with the different surgical modalities is paramount to appropriately managing the needs of these niche patients.

**Figure 3:**
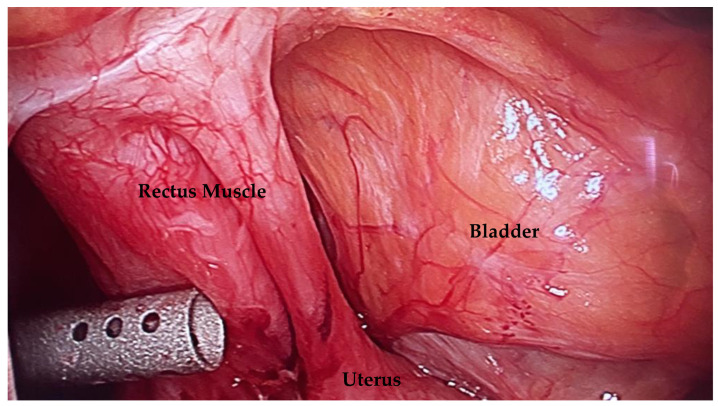
This picture demonstrates dense adhesions between the uterus, rectus muscle, and bladder because the peritoneum was not closed during cesarean delivery. This can lead to more difficult niche repair and hysterectomy.

We also recommend that obstetricians close the peritoneum during cesarean delivery. In our experience, leaving the peritoneum open can lead to severe cases of scar tissue formation and anterior cul-de-sac obliteration, as in Figure 3. The extensive adhesions caused by leaving the peritoneum unrepaired may make it more difficult to reach the vesicovaginal space during niche repair and hysterectomy, if needed, leading to possibly higher complication rates.

Postoperative care requires the monitoring of the patient for symptom resolution and counseling regarding future fertility plans. In general, due to our improved niche repair technique of a two-layer closure without entering the uterine cavity, we recommend patients to wait 6 weeks following repair before attempting conception. However, this timeframe is further individualized depending on the size of the defect.

## 5. Conclusions

Niche should be considered as a differential diagnosis in patients with previous cesarean section that suffer from infertility, pelvic pain, postmenstrual spotting or bleeding. The laparoscopic approach of the niche allows the restoration of normal anatomy, treatment of the endometriosis, as well as adhesive disease that is commonly found with the niche. The outcomes of laparoscopic niche repair are promising and effective in treating symptoms.

We recognize various limitations in our study. Firstly, due to being a surgical referral center, we relied on the evaluations and diagnoses reached by reproductive endocrinology and infertility specialists or other referring physicians. As a result, we do not have the size of the niche and residual myometrium. All we can say is that these patients were symptomatic and alternative treatments had failed. The only remaining option was surgical intervention, which is the reason the patients were referred to our center. We recognize this lack of direct measurement is one of our shortcomings.

It is important to note that our study followed a retrospective design, meaning our findings should be considered clinical observations rather than definitive conclusions. Furthermore, we did not have a control group for comparison purposes. To address this limitation, a future study could include asymptomatic controls who achieved pregnancy naturally.

Despite our longstanding knowledge of the occurrence of niche, this knowledge is not yet widespread among practitioners [6]. Accurate diagnosis is critical in order to appropriately treat this patient population. Patients with a history of prior cesarean deliveries and infertility, pelvic pain or dysmenorrhea may also consider a surgical solution as an option. Surgical intervention for addressing the niche has shown promising results in the treatment of infertility, with minimal complications. Our findings strongly suggest that surgical management of this condition can effectively alleviate or minimize post-menstrual and irregular spotting, leading to outstanding patient satisfaction and acceptance. Moreover, patients have reported remarkable contentment and a notable enhancement in their overall quality of life.

## Figures and Tables

**Table 1:** Baseline characteristics.

Characteristics		Total (*n* = 27)	Percent
Age	≤30	1	3.7%
	31–35	11	40.7%
	>35	15	55.6%
Average BMI		23.1	
Previous LSC with TOE		11	40%
Primary Chief Complaint	Pain	10	37%
	Irregular bleeding or spotting	2	7%
	Infertility	4	14%
	Pain and bleeding	7	26%
	Pain and infertility	3	11%
	Irregular bleeding and infertility	1	3%
Pre-operative IVF attempts		9/17	52%
Pre-operative IUI attempts		8/17	29.6%
Parity	1	20	74%
	≥2	7	26%
History of Smoking		1	3.7%
History of Diabetes Mellitus		2	7.4%
Symptoms	Irregular vaginal bleeding	18	67%
	Pelvic pain	22	81%
	Urinary symptoms	12	44%
	Dysmenorrhea	19	70%
Existing Conditions	Infertility < 1 year	2	7%
	Infertility ≥ 1 year	11	41%
	Previous surgical diagnosis of endometriosis	12	44%
Modality of Diagnosing Niche	Sonogram	19	70%
	Sonohysterogram	3	11%
	MRI	1	3.7%
	Not diagnosed	4	15.3%
Surgical Approach of Repair	Hysteroscopy only	1	3.7%
	Laparoscopy and hysteroscopy	23	85.2%
	Hysterectomy	3	11.1%
Number of Cesarean Deliveries	1	20	74%
	≥2	7	26%

LSC = laparoscopy. TOE = treatment of endometriosis.

**Table 2:** Outcomes after surgical repair of the niche.

Outcomes		Total (*n* = 27)	
Symptoms	Persist	1	3.7%
	Improve	11	40.7%
	Resolved	15	55.6%
Pregnancy Rate *		11/15	73.3%
Live Birth Rate *		9/15	60%
Delivery Route	Vaginal Birth After Cesarean	1	6.6%
	Repeat Cesarean Delivery	8	53.3%
Number of Spontaneous Miscarriages		2	13.3%
Post-Operative Complications	Umbilical Hernia	1	3.7%
	Umbilical Infection	1	3.7%
	Uterine rupture	0	
	Cesarean scar pregnancy	0	

* In patients desiring future pregnancy.

## Data Availability

Data is available by contacting the corresponding author.
